# How can we reduce healthcare costs by using Enhanced Recovery After Surgery more effectively in different groups of gynaecological patients? A single‐centre experience

**DOI:** 10.1111/jep.14196

**Published:** 2024-10-18

**Authors:** Markéta Polková, Peter Koliba, Pavel Kabele, Oľga Dubová, Daniel Hodyc, Magdalena Kolínková Škodová, Michal Zikán, Petra Sládková, Marie Tichá, Tomáš Brtnický

**Affiliations:** ^1^ Department of Gynaecology and Obstetrics, 1st Faculty of Medicine and Bulovka University Hospital Charles University Prague Czech Republic; ^2^ Advance Hospital Analytics Prague Czech Republic; ^3^ Physiotherapy and Medical Rehabilitation Department Bulovka University Hospital Prague Czech Republic

**Keywords:** diagnosis related groups, enhanced recovery after surgery, gynaecologic oncology, gynaecologic surgical procedures, health care costs, outcome assessment

## Abstract

**Introduction:**

The objective of this study was to assess the impact of the Enhanced Recovery After Surgery (ERAS) programme implementation on treatment costs at a university‐type centre, using the DRG scheme.

**Materials and Methods:**

Retrospective analysis of patients' data in a group of 604 individuals enroled in the study. We evaluated three groups of patients according to the ERAS clinical protocol (CP): (1) CP oncogynaecology, (2) CP simple hysterectomy, (3) CP laparoscopy. The study aimed to evaluate the impact on the length of stay (LOS), savings in bed‐days, and the reduction in direct treatment costs. Three parameters—antibiotic consumption, blood derivative consumption and laboratory test costs—were chosen to compare direct treatment costs. The statistical significance of the difference in the observed parameters was tested by a two‐sample unpaired *t* test with unequal variances at the 0.05 significance level.

**Results:**

We analysed data from 604 patients. In all three groups, the length of stay (LOS) was significantly reduced. The most significant reduction was observed in the CP oncogynaecology group, where the LOS was reduced from 11.1 days to 6.8 days (2022) and 7.6 days (2023) compared to 2019 (*p* < 0.05). Furthermore, there was a notable reduction in inpatient bed‐days, which resulted in the capacity being made available to admit additional patients. A statistically significant reduction in direct costs was observed in the group of CP hysterectomy (antibiotic use) and in the CP laparoscopy (laboratory test costs).

**Conclusions:**

The implementation of the ERAS principles resulted in a number of significant positive economic impacts—reduction in the LOS and a corresponding increase in bed capacity for new patients. Additionally, direct treatment costs, including those related to antibiotic use or laboratory testing were reduced. The Czech Republic's acute healthcare system, like the majority of European healthcare systems, is financed by the DRG system. This flat‐rate payment per patient encourages hospital management to seek cost‐reduction strategies. The results of our study indicate that fast‐track protocols represent a potential viable approach to reducing the cost of treatment while simultaneously meeting the recommendations of evidence‐based medicine.

**Abbreviations**
ATB: antibiotics
CNB: Czech National Bank
CP: clinical protocol
CZ‐DRG system: Czech–Diagnosis Related Group
ERAS: enhanced recovery after surgery
ICU: intensive care unit
IHIS CR: Institute of Health Information and Statistics of the Czech Republic
LOS: length of stay

## 1 INTRODUCTION

Enhanced Recovery After Surgery (ERAS) is multimodal perioperative care aimed at achieving early recovery for patients undergoing surgery. This concept of a multimodal approach to the patient referred for surgery was first introduced by Prof. Kehlet in the 1990s.^1^ This programme fully implements and regularly updates perioperative management based on evidence‐based medicine to standardize patient care and mitigate pathophysiological dysfunctions that can arise from surgery and postoperative recovery. Key interventions include adequate preoperative patient education, minimization of preoperative fasting, preoperative administration of carbohydrate solutions, intraoperative euvolemia, standardized opioid‐sparing analgesia, prevention of postoperative nausea and vomiting and early postoperative patient mobilization.^2^, ^3^, ^4^ The aim of these interventions is to reduce postoperative insulin resistance, accelerate the recovery of intestinal motility and reduce the length of hospital stay without a negative impact on the incidence of postoperative complications.^5^ Several publications have already supported this thesis and demonstrated that the introduction of the ERAS programme has led to a reduction in postoperative hospitalisation, a reduction in the incidence of postoperative complications, a reduction in the use of opioid painkillers and a faster functional recovery.^6^, ^7^ Clinical outcomes are most important for patients and their healthcare providers. However, in light of the ever‐increasing costs of healthcare, cost‐effectiveness is also increasingly being discussed and is of general interest to hospital managers, health insurers and politicians alike. It has been shown that the introduction of this protocol into clinical practice does not increase costs but can instead decrease them by shortening the LOS.^8^ However, less attention has been paid to the cost of the treatment required to achieve these outcomes. Compared to, for example, the US health care system, where the cost of treatment is more difficult to ascertain and varies significantly from hospital to hospital,^9^ the Czech health care system is more transparent on this parameter due to the sophisticated system of health insurance companies and the IHIS CR system. Inpatient care in the Czech Republic is covered by the DRG system. Inpatient case payment, where the hospital is reimbursed a flat rate per patient from admission to discharge means the hospital is thus incentivised to improve efficiency by reducing for example, unnecessary tests or shortening the LOS. From published studies, cost reductions have been demonstrated following the implementation of the ERAS programmes in various surgical specialties.^10^, ^11^ From several published studies regarding gynaecological surgery affected by the ERAS programmes, cost savings have been presented as a secondary outcome.^12^, ^13^ However, no study published to date has looked in detail at whether the reduction in treatment costs is mainly due to a reduction in the LOS, freeing up bed capacity for possible additional cases, or a direct reduction in the cost of drugs, medical devices and so forth. In our work, we analyse the savings in both indirect costs of treatment (reduction in the LOS) and direct costs such as consumption of transfusions, antibiotics and the number of laboratory tests carried out.

The following tables summarise the main ERAS recommendations (see Table 1) and a summary of the most important benefits of following them (see Table 2).

**Table 1:** ERAS recommendations.

ERAS	Basic recommendations
Education	Careful informing of the patient about the planned procedure
	Both in oral and written form (leaflet, brochure, etc.)
Preoperative optimization of health status	Smoking and alcohol abstinence for at least 4 weeks before the procedure
	Diabetes—Correction of hyperglycaemia
	Anaemia—Treatment with iron preparations (oral or parenteral)
Prehabilitation	Improving aerobic fitness
	Nutritional interventions
	Psychological intervention
Intestinal preparation	Do not administer during surgery without planned/probable bowel resection
	Consider administration of oral nonabsorbable ATBs during planned bowel resection
Preoperative fasting	Fluids p.o. up to 2 h and food up to 6 h before anaesthesia
	Administration of a carbohydrate solution (e.g., PreOp) up to 2 h before surgery reduces postoperative insulin resistance and should be used routinely
Prevention of thromboembolism	Pharmacological (with low molecular weight or unfractionated heparin)
	Mechanical (compression stockings, DK bandages)
	Apply for procedures lasting longer than 30 min
	Start before surgery, continue throughout hospitalization
	In patients with advanced ovarian cancer and in patients at risk continue pharmacological prophylaxis for 28 days after surgery
Skin preparation	Do not shave with a razor, if necessary remove hair with an electric shaver
Premedication	Not routinely administer long‐acting sedatives
Antibiotic prophylaxis	Intravenously 0–60 min before anaesthesia or 30–60 min before incision
	Cephalosporins first generation
	In obese patients (BMI > 35 or weight > 100 kg) the dose is increased
	Repeat administration according to half‐time (cefazolin after 3 h) and when blood loss >1500 mL
	In bowel surgery, add ATBs with anaerobic spectrum of activity
Standardisation of anaesthetic protocol	Administration of anaesthetics with minimal residual effect for rapid recovery of cognitive function (propofol) is recommended for the management of general anaesthesia
Prevention of hypothermia	Heated pads, blankets and warmed IV solutions
Using drains and probes	Do not use routinely, even for procedures involving bowel resection
	Minimize the insertion of the nasogastric probe, when used for gastric decompression in laparoscopic procedures, remove it at the end of the operation
Infusion treatment	Preference of balanced crystalloid solutions over saline
	Oliguria 20 mL/h corresponds to the response to surgical stress
	Attempt to stop infusion therapy within 24 h after surgery
Prevention of paralytic ileus	Early resumption of per os intake
	Drinking coffee, chewing gum
	Reduce the amount of opioids
Postoperative nutrition	Early realimentation, sipping
	High‐protein diet
Urine derivation	Urinary catheter extraction within 6 h after surgery
Postoperative pain management	Preference of epidural anaesthesia/analgesia
	Take advantage of the synergistic action of NSAIDs, paracetamol, gabapentin, dexamethasone
	Reduction of opioid consumption
Early verticalization	Verticalization on the day of surgery
	Four hours out of bed first post‐op. day
	Eight hours out of bed second post‐op. day
Discharge to outpatient care	Clearly defined discharge criteria
	Inform the patient and family members about possible complications and their symptoms

*Note*: Adapted from Guidelines for Preoperative and Intraoperative Care in Gynecologic/Oncologic Surgery, Guidelines for perioperative care in gynecology/oncology: Enhanced Recovery After Surgery (ERAS®) Society Recommendations Part I and Part II, Enhanced Recovery After Surgery (ERAS) Society recommendations‐2019 update, Enhanced recovery after surgery (ERAS®) society guidelines for gynecologic oncology: Addressing implementation challenges‐2023 update.

**Table 2:** Benefits of ERAS.

Benefits of ERAS	Description
Reduction of postoperative complications	ERAS minimizes complications associated with surgical procedures through strict management of factors such as preoperative hydration, nutrition and pain control. It reduces the risk of infections, thromboses and other complications.
Reduction in length of hospital stay	The ERAS programme promotes faster mobilisation of patients and introduces procedures that reduce the need for long hospital stays. It reduces healthcare costs and increases patient satisfaction.
Improved pain control	ERAS uses a multimodal approach to pain control, incorporating different types of analgesics and techniques to minimize the use of opioids, which reduces side effects and improves patient well‐being
Improved nutrition and metabolic support	Optimizing nutrition before and after surgery is crucial. The programme includes preoperative carbohydrate loading and early introduction of oral intake after surgery, which improves healing and reduces muscle catabolism.
Psychological support and education	Educating patients about the surgery and recovery process helps them feel less stressed and anxious, which helps them recover better

*Note*: Adapted from Guidelines for Preoperative and Intraoperative Care in Gynecologic/Oncologic Surgery, Guidelines for perioperative care in gynecology/oncology: Enhanced Recovery After Surgery (ERAS®) Society Recommendations Part I and Part II, Enhanced Recovery After Surgery (ERAS) Society recommendations‐2019 update, Enhanced recovery after surgery (ERAS®) society guidelines for gynecologic oncology: Addressing implementation challenges‐2023 update.

### 1.1 Comparison of the health system in the Czech Republic with the systems of other European Union countries

Health systems in the Czech Republic and most EU countries are similar in that they mostly operate on the principle of public health insurance, which is compulsory and financed by contributions from employees, employers and the state. All these systems ensure wide access to healthcare for all citizens, regardless of their income. Most European countries, including the Czech Republic, use the DRG (Diagnosis‐Related Group) system for reimbursement of hospital care, which allows for efficient allocation of funds based on diagnoses and patient needs. Healthcare in these systems is primarily organised by public or private entities that are regulated by the state. The systems emphasize accessibility, quality and efficiency of health care.^14^

### 1.2 Comparison of the Czech healthcare system with the system in the United States

The financing and functioning of health systems in the Czech Republic and the United States differ in many aspects (see Table 3). The health system in the Czech Republic is based on public health insurance with a high degree of solidarity and equal access to care. Financing is mainly provided by compulsory levies and direct payments by patients are relatively low. In the United States, the system is much more based on private insurance with higher direct payments and significant inequalities in access to care. Healthcare costs are generally much higher in the United States than in the Czech Republic. On the other hand, both health systems share the same basic principle of financing by using DRG (diagnosis related group) (see Table 4). While both systems use DRGs to pay for hospital care, in the Czech Republic, the system is less detailed and adapted to local conditions, while in the United States the system is very complex, with a high level of detail and specificity, especially in the Medicare programme. In the United States, there is also a higher administrative burden associated with the use of DRGs, reflecting the complexity of the system.^15^, ^16^, ^17^

**Table 3:** Health systems comparison.

Healthcare systems comparison		
	Czech Republic	United States of America
Funding	Public, from compulsory health insurance	Mixed, private insurance, public programmes, direct payments
Ratio of direct payments	Low	High
Main sources of funding	Compulsory health insurance	Private insurance, Medicare, Medicaid, direct payments
Healthcare costs	Relatively low	The highest in the world
Role of insurance companies	Public health insurance companies (VZP, others)	Mostly private insurance companies, public programmes for selected groups
Access to healthcare	Equal access, same level of care for all	Depends on the quality and type of insurance, unequal access
Public programmes	The government pays for children, pensioners and the unemployed	Medicare (seniors), Medicaid (low‐income)
Co‐payments and fees	Low	High

**Table 4:** DRG systems comparison.

DRG systems comparison		
	Czech Republic	United States of America
Implementation of the DRG	Phased in, now key to funding	One of the first DRG systems, originally for Medicare
Adaptation to local conditions	Yes, the system is adapted for the Czech healthcare system	A very specific and complex system, especially for Medicare
Purpose of the use	Mainly for reimbursement of acute hospital care	Used for Medicare and adapted by private insurers
Administrative burden	Relatively lower	Higher administrative requirements and complexity
Functioning of the system	Continual improvement and adaptation	A stable but comprehensive system with a focus on cost control

The economic as well as the clinical motivations are basically the same in all countries using the DRG system to pay for acute inpatient care. Thus, in most European countries, as well as in the United States, it is economically advantageous from the provider's point of view to shorten hospital stays and rationalise laboratory, drug or transfusion costs. Furthermore, this motivation is aligned with the clinical goal of recovering the patient as quickly as possible, as per the ERAS concept.

## 2 MATERIALS AND METHODS

**Figure 1:**
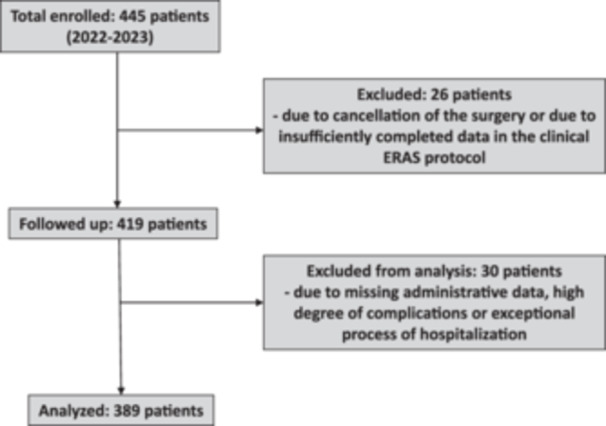
CONSORT diagram.

A total of 604 patients were analyzed in the study. The study included all patients (without randomisation) between the ages of 18 and 82 who underwent primary oncogynaecological surgery, simple hysterectomy or laparoscopy during the period indicated below. We implemented the ERAS protocol at our institution in 2022 and enroled a total of 188 patients in the ERAS programme (period 03–09/2022), 163 patients were analysed. In 2023, a total of 257 patients were enroled in the programme (period 01–09/2023), 226 patients were analysed. A total of 389 patients in 2022/2023 were analysed (see CONSORT diagram, Figure 1).

**Figure 2:**
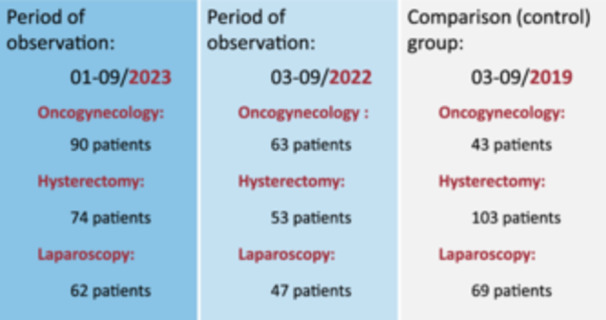
Patients analyzed in the study.

In contrast, a comparison group was established in 2019 (period 03–09/2019), where 215 cases met similar criteria (see Figure 2). Thus, this is a retrospective study in which we evaluated three groups of patients according to the ERAS clinical protocol (hereafter referred to as CP): (1) CP oncogynaecology, (2) CP simple hysterectomy, (3) CP laparoscopy. The oncogynaecology group underwent surgery for the diagnosis of ovarian cancer, endometrial cancer and cervical cancer. In the hysterectomy group the following procedures were performed: total laparoscopic hysterectomy, laparoscopically assisted vaginal hysterectomy, vaginal and abdominal hysterectomy. The laparoscopy group included procedures such as adnexectomy, salpingectomy and ovarian cyst resection.

### 2.1 2023 data cleaning methodology

The 2023 data were further cleaned of duplicates in the data (one case), cases assigned to the CZ‐DRG group not matching the gynecological procedure or with another surgery on a different day (nine cases), cases not matching the diagnosis or surgical approach (three cases), cases that could not be paired to the administrative data (four cases). In addition, cases with higher rates of complications and comorbidities that did not occur in the other comparison groups were excluded (three cases).

### 2.2 2022 data cleaning methodology

The 2022 data were further adjusted for outlier cases due to length of hospital stay (using a threshold of 2x the upper trimpoint for the CZ‐DRG group) and cases with reoperation (two cases), and cases not falling within the 03–09/2022 period (two cases), cases assigned to the CZ‐DRG group not matched to a gynecologic procedure or with another surgery on a different day (three cases) and cases that could not be paired to administrative data (three cases).

### 2.3 Selection of the 2019 comparison group and data cleaning methodology

For the comparison (control) group, the year 2019 was selected because 2020 and 2021 were affected by the COVID‐19 pandemic. Cases from 2019 were paired to the 2022 data according to a similar structure with respect to the combination of procedure, principal diagnosis and CZ‐DRG classification. The 2019 data was further adjusted for outlier cases due to age—excluding cases under 18 years and aged 83 years and over (to maintain a similar age composition compared to 2022 and 2023)—and outlier cases due to length of hospital stay (using a threshold of 2x the upper trimpoint for the CZ‐DRG group). Additionally, cases with higher rates of complications and comorbidities that did not occur in the 2022/2023 data, as well as cases not falling within the same observation period in 2022/2023 were excluded.

In our view, the exclusion of patients from the study for the reasons mentioned above led to more precise and reliable results. Given the size of the cohort, the number of patients was relatively small and the final impact on the results (if any) was, in our opinion, positive.

To calculate bed‐day savings, data were prorated to a full 12 months of the year for all three study groups. We chose three parameters to compare direct treatment costs: antibiotic consumption, blood derivative consumption and laboratory testing costs. Due to the increase in the price of direct treatment costs, the prices of antibiotics, blood derivatives, and laboratory tests were compared at 2019 levels in all three observed groups.

### 2.4 Statistical analyses

The statistical significance of the difference in the observed parameters was tested by a two‐sample unpaired *t* test with unequal variances at the 0.05 significance level.

## 3 RESULTS

The retrospective study analysed a total of 604 patients. Namely, 196 patients in the oncogynaecology CP, 230 patients in the hysterectomy CP, and 178 patients in the laparoscopy CP (see Figure 2).

### 3.1 Length of hospital stay (LOS)

**Figure 3:**
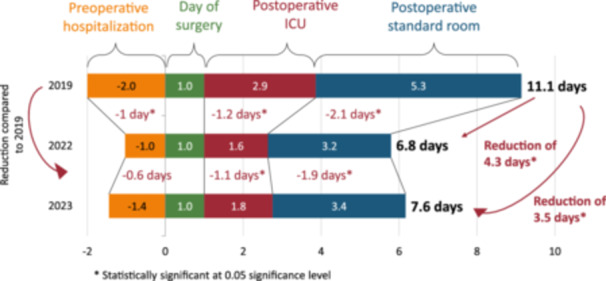
The reduction of LOS in CP oncogynecology. CP, clinical protocol; LOS, length of stay.

In the oncogynaecology group, the total LOS was reduced from 11.1 days to 6.8 days (2022) and 7.6 days (2023) compared to 2019. There was also a standardization of the length of preoperative hospitalization from 2.0 days (2019) to 1.0 (2022) and 1.4 (2023), respectively. In postoperative care, there was a reduction in the ICU LOS from 2.9 (2019) to 1.6 (2022) and 1.8 (2023), respectively (*p *< 0.05) (see Figure 3).

**Figure 4:**
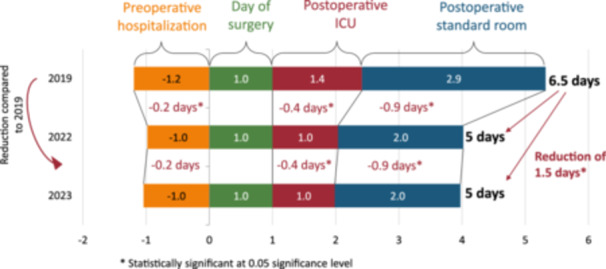
The reduction of LOS in CP hysterectomy. CP, clinical protocol; LOS, length of stay.

In the group—CP hysterectomy, there was a reduction in the total LOS from 6.5 days (2019) to 5.0 days (2022, 2023) (*p *< 0.05). There was a standardization of the preoperative LOS and a reduction in the postoperative ICU and standard room stay (see Figure 4).

**Figure 5:**
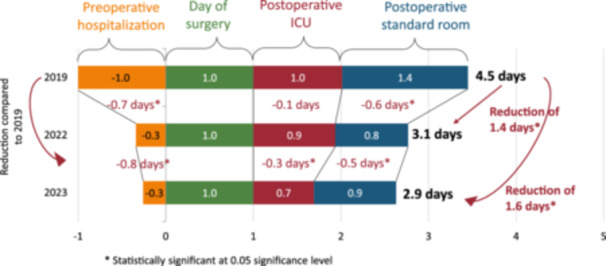
The reduction of LOS in CP laparoscopy. CP, clinical protocol; LOS, length of stay.

In the group—CP laparoscopy, the LOS was reduced from 4.5 days (2019) to 3.1 days (2022) and 2.9 days (2023), respectively (*p *< 0.05). The majority of patients were admitted on the day of surgery, and in 2023, one‐third of patients were not admitted to the ICU after surgery at all (see Figure 5).

### 3.2 Hospital bed‐day saving

In the group—CP oncogynaecology, the ICU stay was reduced by 1.1 days and the standard room stay by 2.4 days (comparison of 2019 and 2023). Therefore, the annual inpatient savings calculated on the number of cases in 2023 (annualized) corresponded to a total of 132 days in the ICU and 288 days in a standard room. The potential number of cases with oncogynaecological diagnosis that could be treated additionally was calculated to be 73 per year in the ICU and 59 in a standard room. Due to the annual savings in the ICU and standard room, approximately 59 additional patients could be admitted for oncogynaecology procedures under the current length of stay (2023) as a result of the freed‐up capacity.

In the group—CP hysterectomy, there was a total annual saving of 40 ICU days and 109 bed days in a standard room. With this reduction, approximately 36 additional patients could be admitted for the simple hysterectomy procedure under the current LOS (2023).

In the group—CP laparoscopy, the calculated annual savings were 25 bed‐days in the ICU and 108 bed‐days in a standard room. This saving allows for approximately 36 additional patients undergoing laparoscopic procedures over the current LOS (2023).

### 3.3 Cost savings on treatment days

The average cost in 2023 per patient per day in our ICU was €829* and €259* for a standard room.

In the group—CP oncogynaecology, an annual saving of 132 bed days in the ICU and 288 bed days in a standard room was calculated. The calculated annual financial saving in the ICU was €109,481* and in a standard room €74,651*. The total potential calculated annual savings in the care of oncogynaecological patients was €184,132*.

In the group—CP hysterectomy, an annual saving of 40 days in the ICU or 109 days in a standard room was calculated. The calculated potential annual financial savings were €33,314* in the ICU and €28,371* in a standard room. The total potential calculated annual savings in the care of patients undergoing simple hysterectomy was €61,685.

In the group—CP laparoscopy, an annual saving of 25 days in the ICU or 108 days in a standard room was calculated. The calculated potential annual financial savings were €16,679* in the ICU and €27,994* in a standard room. The total potential calculated annual savings in the care of patients undergoing simple laparoscopy was €44,673*.

* (Currency conversion from Czech crown to Euro according to the current exchange rate of the Czech National Bank [CNB] as of 20 April 2024, http://www.cnb.cz/en/).

### 3.4 Direct costs of transfusions, antibiotics and laboratory tests

In terms of direct costs, we decided to analyse the trend in the administration of antibiotics, transfusion derivatives and the cost of laboratory tests.

In the group—CP oncogynaecology, the average cost of laboratory tests, antibiotics, and transfusions was lower in both 2022 and 2023 compared to 2019. However, the results were not statistically significant at the 0.05 significance level.

**Figure 6:**
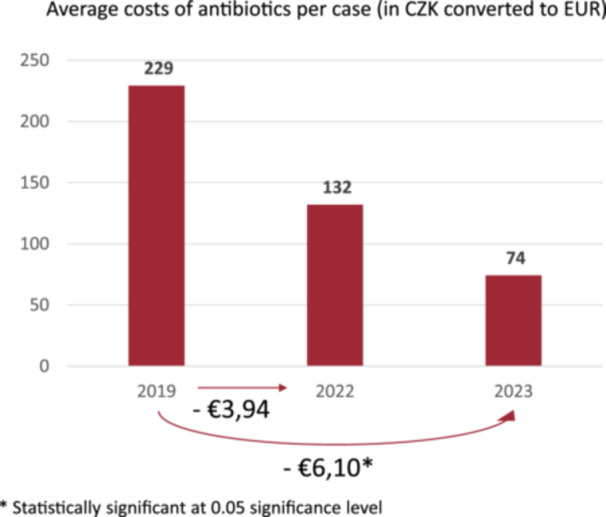
Reduction in costs in CP hysterectomy—antibiotic administration. CP, clinical protocol.

In the group—CP hysterectomy, the reduction in costs was statistically significant only for antibiotic administration, and this was for the 2023 versus 2019 comparison (see Figure 6). The results were not statistically significant for laboratory testing and transfusion costs.

**Figure 7:**
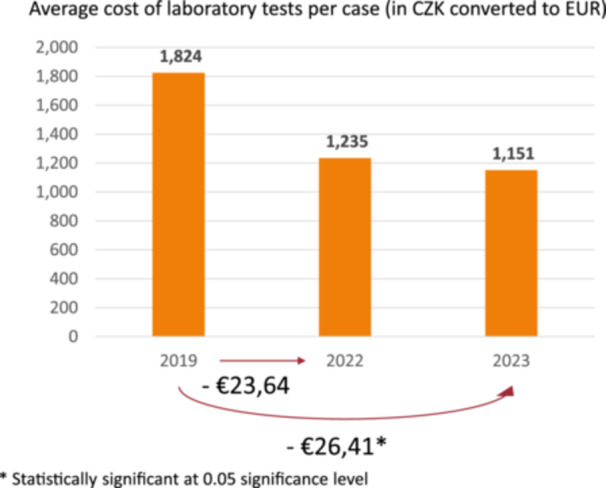
Reduction in costs in CP laparoscopy—laboratory tests.

In the group—CP laparoscopy, there was a statistically significant reduction in the cost of laboratory testing in 2023 compared to 2019 (see Figure 7). No statistically significant reduction in financial costs was found in the remaining parameters monitored (antibiotics and transfusion).

## 4 DISCUSSION

Many studies have already demonstrated the positive effect of implementing the ERAS programme into routine practice. Most publications are traceable to surgical disciplines, especially in colorectal surgery, and these studies report a reduction in the LOS after the ERAS implementation of between 0.5 and 3.5 days on average.^18^, ^19^, ^20^, ^21^ However, even in gynaecological surgery, there are several papers reporting a reduction in the LOS, ranging from 5.4 to 7.3 days on average.^13^, ^22^, ^23^ These are in most cases retrospective studies, which correlate in the sense of the LOS shortening with our results. The retrospective approach appears to be appropriate and valid for tracking the impact of ongoing implementation processes over time, both before and after staff was introduced to each ERAS recommendation. The implementation of the ERAS programme at our workplace has influenced and significantly changed the approach and mind‐set of the entire medical and nursing team towards the patient, from the outpatient examination to the day of discharge. Consequently, the staff automatically reduced preoperative fasting by administering carbohydrate solutions, reduced preoperative bowel preparation and so forth, even in patients who were not enroled in the ERAS programme. It would then be difficult to prospectively separate cohorts of patients with and without the ERAS recommendations. A prospective study would then only be possible assuming a multicentre study design in which the control site would have no prior experience with the ERAS recommendations. In our study, in addition to the reduction in the LOS, we demonstrated significant cost savings. Both in indirect costs, in the sense of shortening the LOS and freeing up bed capacity for other patients, and in direct cost savings (financial savings in the operation of the facility). In the case of indirect costs, the savings are both immediate, that is shortening the LOS reduces the cost of running the unit, and hypothetical, that is the vacant bed after the patient is discharged may not necessarily be taken up or may be occupied with a delay. In terms of direct costs, we focused on the consumption of antibiotics, transfusion derivatives and the cost of laboratory tests. Among the monitored parameters, we demonstrated statistically significant savings in antibiotic consumption in the group of patients undergoing a simple hysterectomy and also savings in laboratory testing costs in the group of patients undergoing an uncomplicated laparoscopic procedure. The saving in direct costs is a clearly defined economic parameter compared to the reduction in the LOS. As we only collected data on patients during hospitalisation, we did not assess the potential savings from reducing readmissions. The strong point of our project is the comprehensive view of the change in the economics of a single institution after the introduction of the ERAS programme, both in the LOS development and in the savings in direct treatment costs for more than 600 patients operated on for both benign and malignant gynaecological diagnosis. As already mentioned, acute inpatient care in the Czech Republic is covered by the DRG system, that is payment per hospital case, in which the hospital is charged a flat rate for the patient from admission to discharge. Hospital management is thus motivated to enhance efficiency by reducing unnecessary examinations and tests or shortening the LOS. This is why there has been a demand for fast‐track concepts in the Czech Republic in recent years. Thus, it can be emphasized that this paper and its result is relevant from a cost‐effectiveness perspective in every country where DRGs are used—which is almost every country in Europe. Although successful implementation of fast‐track protocols requires considerable effort on the part of hospital staff and careful monitoring of protocol adherence and outcomes, as this publication shows, it can lead to financial savings while ensuring a higher quality of care.

## 5 CONCLUSION

We have demonstrated cost savings particularly in the reduction of the LOS. However, reduced direct costs, such as antibiotic consumption and laboratory costs, have also contributed significantly. Ultimately, the implementation of the ERAS protocol at our institution has demonstrated that better quality medicine based on best clinical practice can be implemented, reducing the cost of care while freeing up bed capacity for additional patients. The results of this study also have significant international overlap. The implementation of the ERAS protocol provides an example of how improvements in clinical practice and efficiency can lead to significant benefits for health systems worldwide. In countries that use a DRG system, implementation of the ERAS protocol has the potential to improve the quality of care and efficiency of hospital resource use, which is crucial in the context of global efforts towards sustainable and cost‐effective health systems. The results of this study support the idea that improvements in clinical practice and protocols can yield both quality and economic benefits across health systems worldwide.

## CONFLICT OF INTEREST STATEMENT

The authors declare no conflict of interest.

## ETHICS STATEMENT

Approval from the Ethics Committee of the University Hospital Bulovka (registration number 10875/EK‐Z) was granted on 8 June 2023.

## Data Availability

Research data are not shared.
